# Arthropod Borne Disease: The Leading Cause of Fever in Pregnancy on the Thai-Burmese Border

**DOI:** 10.1371/journal.pntd.0000888

**Published:** 2010-11-16

**Authors:** Rose McGready, Elizabeth A. Ashley, Vanaporn Wuthiekanun, Saw Oo Tan, Mupawjay Pimanpanarak, Samuel Jacher Viladpai-nguen, Wilarat Jesadapanpong, Stuart D. Blacksell, Sharon J. Peacock, Daniel H. Paris, Nicholas P. Day, Pratap Singhasivanon, Nicholas J. White, François Nosten

**Affiliations:** 1 Shoklo Malaria Research Unit (SMRU), Mae Sot, Tak, Thailand; 2 Mahidol-Oxford Tropical Medicine Research Unit (MORU), Mahidol University, Bangkok, Thailand; 3 Centre for Tropical Medicine, Churchill Hospital, Oxford, United Kingdom; 4 Imperial College NHS Trust, London, United Kingdom; 5 Department of Medicine, University of Cambridge, Addenbrooke's Hospital, Cambridge, United Kingdom; University of California San Diego School of Medicine, United States of America

## Abstract

**Background:**

Fever in pregnancy is dangerous for both mother and foetus. In the 1980's malaria was the leading cause of death in pregnant women in refugee camps on the Thai-Burmese border. Artemisinin combination therapy has significantly reduced the incidence of malaria in the population. The remaining causes of fever in pregnancy are not well documented.

**Methodology:**

Pregnant women attending antenatal care, where weekly screening for malaria is routine, were invited to have a comprehensive clinical and laboratory screen if they had fever. Women were admitted to hospital, treated and followed up weekly until delivery. A convalescent serum was collected on day 21. Delivery outcomes were recorded.

**Principal Findings:**

Febrile episodes (n = 438) occurred in 5.0% (409/8,117) of pregnant women attending antenatal clinics from 7-Jan-2004 to 17-May-2006. The main cause was malaria in 55.5% (227/409). A cohort of 203 (49.6% of 409) women had detailed fever investigations and follow up. Arthropod-borne (malaria, rickettsial infections, and dengue) and zoonotic disease (leptospirosis) accounted for nearly half of all febrile illnesses, 47.3% (96/203). Coinfection was observed in 3.9% (8/203) of women, mostly malaria and rickettsia. Pyelonephritis, 19.7% (40/203), was also a common cause of fever. Once malaria, pyelonephritis and acute respiratory illness are excluded by microscopy and/or clinical findings, one-third of the remaining febrile infections will be caused by rickettsia or leptospirosis. Scrub and murine typhus were associated with poor pregnancy outcomes including stillbirth and low birth weight. One woman died (no positive laboratory tests).

**Conclusion/Significance:**

Malaria remains the leading cause of fever in pregnancy on the Thai-Burmese border. Scrub and murine typhus were also important causes of fever associated with poor pregnancy outcomes. Febrile pregnant women on the Thai-Burmese border who do not have malaria, pyelonephritis or respiratory tract infection should be treated with azithromycin, effective for typhus and leptospirosis.

## Author Summary

Fever during pregnancy can be harmful for the mother and the infant. In resource poor settings health workers have very few field-based tests that help them identify the cause of infection. This study examined the causes of fever in pregnant women using laboratory support that is typically unavailable to most women living in the tropics. On the Thai-Burmese border there has been a great reduction in malaria in the last 20 years. However malaria remained the leading cause of fever in pregnancy in this study conducted between 2004 and 2006. Urinary tract infection was also a common cause of fever as it is in resource rich countries. Other diseases transmitted by mosquitoes (dengue), ticks (scrub and murine typhus), or rodents (leptospirosis) were common. Scrub and murine typhus were associated with stillbirth and low birth weight. Microscopy remains the most useful tool in the field for the diagnosis of fever in pregnant women. Leptospirosis, dengue and rickettsial infections require improved field-based diagnostic tools to ensure that women receive appropriate antibiotic therapy.

## Introduction

Febrile illness in pregnancy increases the risk of maternal death and infant morbidity and mortality. Relatively little is known about the etiology of fever in pregnancy in resource poor populations. It is associated with poor pregnancy outcomes including miscarriage, stillbirth and premature delivery [1].

In 1984-86 malaria was the major cause of medical consultation in the newly arrived refugee population and the leading cause of maternal death on the Thai-Burmese border. An estimated 1% of all pregnant women died per year from malaria, a rate equivalent to a maternal mortality of 1,000 per 100,000 live births [2]. This is an area where multi-drug resistant strains of *P. falciparum* are prevalent [3] and there is currently no safe and effective drug that can be offered to pregnant women for antimalarial prophylaxis. The only proven alternative in this area is antenatal clinics which provide weekly screening by malaria smear for early detection and treatment [2]. There are few diagnostic tools for malaria smear negative febrile patients. Without a confirmed diagnosis providing appropriate antimicrobials is difficult. In a prospective study of pregnant women and their infants (n = 1,495) on the Thai-Burmese border, maternal febrile illness of any cause was associated with a 2.5 (95%CI 1.3–5.0) fold increased risk of neonatal death [4]. A better understanding of the causes of febrile illness in pregnant women in rural tropical areas could reduce maternal and neonatal morbidity and mortality. This pilot survey aimed to identify the organisms responsible for fever in pregnant women on the North-Western Thai-Burmese border.

## Methods

### Ethics statement

The patient information sheet and consent form were available in Karen and Burmese languages. Willing participants signed (literate) or provided a thumb print (illiterate) on the consent form. Approval for the study was granted by the Oxford Tropical Research Ethics Committee, UK (#013-03).

### Study sites

**Figure 1:**
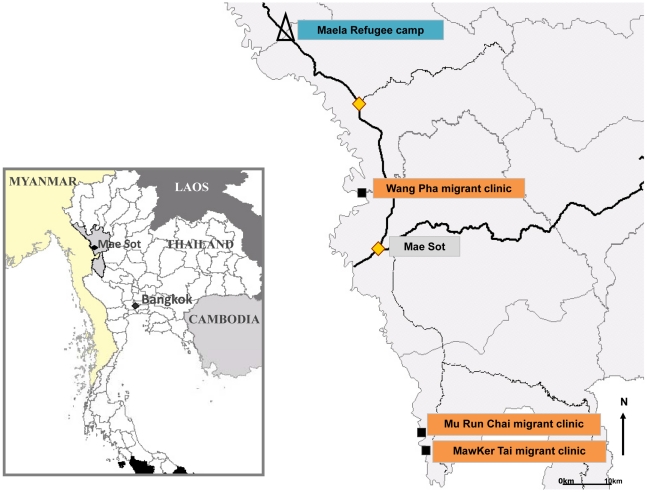
Map of the study area including Maela Refugee camp and migrant clinic sites.

The Shoklo Malaria Research Unit encouraged all pregnant women to attend weekly antenatal clinics for active screening for malaria. In Maela Refugee camp five antenatal clinics were attended by approximately 800 women per week and in three migrant sites (Wang Pha, Mu Run Chai and MawKer Tai) four antenatal clinics were attended by approximately 300 women per week (Figure 1). Displaced persons from Burma reside in a semi-open refugee camp (Maela) in Thailand and have access to a basic food ration supplied by a consortium of charities and health care provided primarily by Aide Médicale Internationale. Migrant women are mobile and mostly find agricultural work (on either side of the border) and have limited access to health care facilities. In 2005 the seroprevalences of HIV, 0.4% (95%CI 0.1–1.4), syphilis 0.4% (95%CI 0.1–1.2) [5] and other sexually transmitted infections in refugee women were low (unpublished data). At the time of this study an opt-in prevention of mother to child transmission system of HIV testing was available to pregnant women in the refugee camp and commenced in migrant women in August 2008.

### Sample size

This study used convenience sampling to investigate the cause of fever in pregnant women. Enrolment was limited to 203 pregnant women attending SMRU antenatal clinics based on cost and time restrictions. Enrolment to the study was from May 2004 to January 2006.

### Inclusion criteria

Pregnant women with confirmed fever (aural temperature >37.5°C), regardless of gestation, who agreed to hospitalization (although the clinical condition did not always necessitate this) and gave written informed consent could be included in the study. Exclusion criteria were enrolment to the malaria study running concurrently or prior enrolment to this fever study i.e. individuals were not recruited to more than one study nor could they be enrolled more than once.

Women with uncomplicated *P.falciparum* malaria in the 2^nd^ or 3^rd^ trimester of pregnancy were enrolled into a treatment study reported elsewhere [6]. If they declined to participate in the malaria study they were offered entry to the simpler fever study which had less strict follow-up requirements.

### Laboratory procedures on admission

A total of 8.5 mL of whole blood was taken. Laboratory investigations included malaria thick and thin films (if these were not already available from antenatal clinics), complete blood count, blood culture of 5 mL blood into BacT/ALERT FA aerobic blood culture bottles, rapid immunochromatographic test for scrub typhus (Panbio Pty LTd., Brisbane, Australia), leptospirosis (Biomerieux), and dengue (Panbio); serology and polymerase chain reaction (PCR) of buffy coat for scrub typhus and whole blood for murine typhus. If the woman had a positive rapid immunochromographic test for scrub typhus or leptospirosis then an additional 5 mL was taken for *in vitro*-culture. A mid-stream urine sample was tested by urine dipstick (Combur7), microscopy and culture. In women with persistent cough acid fast bacilli were examined for by microscopy of early morning sputum samples stained with Ziehl-Neelsen stain.

### Laboratory methods and diagnostic definitions

#### Malaria

Thick and thin malaria smears were stained with Giemsa and examined under oil immersion. Smears were only declared negative after 200 fields were read. Uncomplicated malaria was defined as an asexual parasitaemia with less than 4% infected red blood cells in the absence of signs of severe malaria [7]. Hyperparasitaemia was defined by a parasitaemia of ≥4% infected RBC. Anaemia was defined by a haematocrit <30%, and severe anaemia by a haematocrit <20%.

#### Bacteriological culture

Blood samples were inoculated aseptically into blood culture BacT/ALERT FA bottles (BioMérieux, Durham, North Carolina) containing 30 ml of media, activated charcoal, and an internal sensor that detects carbon dioxide as an indicator of microbial growth. Blood culture bottles were vented (using BCB Vent/Sub units; Difco, West Molesey, Surrey, UK) immediately and incubated unshaken in air for seven days at 37°C. Gram stains were performed on smears prepared at 12–24 hours and 36–48 hours, or when the sensor indicated growth.

Ten mLs of urine were collected into a sterile container and transported to Mae Sot on ice. Quantitative urine culture was performed by using a sterile loop to inoculate 0.001 ml urine onto a 5% sheep blood agar and a MacConkey agar plate (Oxoid, Basingstoke, Hampshire, UK). All plates were incubated in air at 35°C and read at 24 and 48 hours for bacterial identification and colony counts. Isolates from specimens containing a single bacterial colony type at greater than or equal to 10^5^ colony forming unit (cfu)/ml were identified and antimicrobial susceptibility tests performed. Single colony types between 10^4^ cfu/ml and 10^5^ cfu/mlwere characterized if symptoms and signs suggested infection. Cultures were considered contaminated if more than 1 organism or non-pathogens were isolated. Pyelonephritis was diagnosed if systemic symptoms such as fever, chills, nausea, vomiting, flank pain or costovertebral angle tenderness were accompanied by a positive urine culture, or if there was a valid reason for culture negativity e.g. antibiotics in the past 72 hours. A positive urine culture in the absence of symptoms of urinary tract infection was defined as an asymptomatic urinary tract infection.

#### Rickettsial disease

Acute murine typhus (*Rickettsia typhi*) and scrub typhus (*Orientia tsutsugamushi)* infections were defined as patients having positive PCR results and/or *in vitro* isolation of *Rickettsia spp* and/or positive reference serology (a 4-fold rise in IgM and/or IgG antibody titre between acute and convalescent serum samples measured by indirect immunofluorescence assay (IFA) [8], [9]). Isolation of *O. tsutsugamushi* and *R. typhi* was attempted in vitro from buffy coats derived from EDTA-treated blood of patients, using a method described previously [10].

Two real-time PCR assays based on the 47 kDa [11] based on intercalating SYBR green [12] and *groEL* [13] gene targets for scrub typhus and ompB [14] for murine typhus were performed on buffy coat samples taken at presentation in serologically positive women. All PCR assays were performed in triplicate in three individual runs.

#### Leptospirosis

Leptospirosis was confirmed by the microscopic agglutination test (MAT) and/or isolation *in vitro* of Leptospira organisms. *Leptospira* culture on the day of admission was performed using blood from the heparin sodium tube (Heparin Leo, Leo Pharma, UK) placed into Ellinghausen McCullough Johnson Harris (EMJH) medium (Difco) supplemented with 3% rabbit serum and 0.1% agarose, as described previously [15]. MAT was performed by the WHO/FAO/OIE Collaborating Centre for Reference & Research on Leptospirosis, Australia [16] using a panel of antigens representing both ubiquitous and locally prevalent serovars. A positive MAT was taken as a 4-fold rise in titer between acute and convalescence samples taken after the onset of symptoms.

#### Dengue

Convalescent samples were screened for dengue virus IgM antibodies using the Panbio IgM antibody capture ELISA (Panbio Pty Ltd. Brisbane, Australia). In patients with positive convalescent samples (>11 Panbio units), paired acute samples were tested retrospectively using the same assay. Following the introduction of the Panbio NS1 antigen detection ELISA (Panbio Pty Ltd., Brisbane, Australia), which detects dengue virus specific antigen in the first 7 days of illness [17], the acute specimens were also tested for the presence of NS1 antigen to provide further evidence of dengue infection.

#### Acute respiratory infections and other diseases

These were diagnosed by clinical symptoms and signs and available laboratory results. Pharyngitis, tonsillitis and sinusitis were included in upper respiratory infection and all women with clinical signs consistent with bronchitis or pneumonia included in lower respiratory tract infection. No radiology was available.

#### Acute undifferentiated febrile illness

For the purpose of this study, fever of any duration with no specific identifying clinical symptoms and signs, and negative diagnostic tests were assigned the diagnosis of acute undifferentiated febrile illness by the investigator after all information from inpatient charts were reviewed.

#### Coinfection

Coinfection was defined as conclusive laboratory evidence for more than one infection.

### Clinical procedures

On admission women were admitted to hospital and had a medical and obstetric history and physical examination by a physician and a midwife. Patients' symptoms were screened using a standardized checklist. Treatment was initiated according to the results of the clinical examination and initial laboratory results (including rapid tests). Results of positive blood and urine culture at 48 hours were communicated to the physician on duty and treatment altered accordingly. PCR, serology, scrub typhus and leptospirosis culture were not available on time to influence clinical management.

Patients were reviewed at least daily and the symptom checklist revised. Vital signs were recorded every 6 hours. Body temperature was measured by an aural thermometer with disposable covers (Genius). Fever clearance time was defined as the time, from onset of treatment, to the first time the aural temperature dropped below 37.5°C and stayed at or below 37.5°C for 24 hours.

### Follow-up of pregnant women: treatment failure and fever recurrence

After discharge patients were followed up weekly at their regular antenatal clinic. The symptom checklist and obstetric examination were repeated weekly for 6 weeks. A convalescent serum sample was taken as close to day 21 as possible. All women were encouraged to attend the unit for supervised delivery with trained midwives, although women traditionally deliver at home in this region.

### Infant follow-up

Gestational age was assessed by ultrasound (≤24 weeks gestation) or by the Dubowitz exam [18] in women with a late, or no ultrasound. Pre-term delivery was defined as a gestational age less than 37 weeks and miscarriage as delivery before 28 weeks gestation. Birth weight was assessed in all infants that were seen after birth (Electronic SECA medical scales, precision 10 g) but analysis for birth outcomes was restricted to those weighed in the first 72 hours of life. Low birth weight was defined by a birth weight <2500 g. Where possible all infants were examined again at 1 month of age.

### Analysis

The main outcome measure was the fever diagnosis. Secondary outcomes included the rate of concomitant infection, pregnancy outcomes including maternal death, abortion, stillbirth, congenital abnormality, birth weight, gestational age and neonatal death. The Mann-Whitney *U* test was used for nonparametric comparisons and Student's *t* test or one-way analysis of variance was used for parametric comparisons. Proportions were examined using χ^2^ test with Yates' correction or by Fisher's exact test. A logistic regression model was used to determine adjusted odds ratio (OR) (95% confidence intervals) for risk factors (gestational age, febrile illness, gravidity and smoking) for low birth weight (LBW). Data were described using the statistical program SPSS for Windows (SPSS Benelux inc., Gorinchem, Netherlands).

## Results

**Figure 2:**
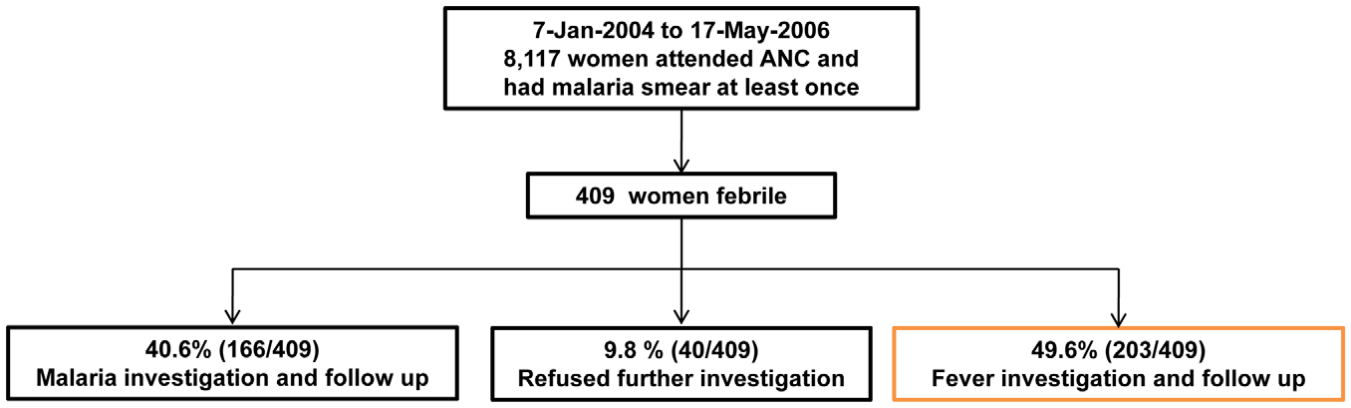
Patient flow.

From 7-Jan-2004 to 17-May-2006 a total of 8,117 pregnant women attended antenatal care. Fever episodes were documented in 5% (409/8,117) of women who had a total of 438 episodes of fever with a median [range] of 1 [1]–[4] episodes per woman. The majority of women had malaria 55.5% (227/409), followed by non-malarial illness 41.8% (171/409) with few women having both, 2.7% (11/409). Amongst the women with fever 49.6% (203/409) participated in the fever etiology study, 40.6% (166/409) participated in the malaria treatment study and 9.8% (40/409) refused to enter any study but received full treatment at the clinic (Figure 2).

The malaria study conducted concurrently raised the possibility of biased selection to the fever study. The characteristics of the women were summarized by malaria and non-malaria fever (Table 1). A significantly greater proportion of women with malaria were: migrants, smokers, and presented earlier in pregnancy than non-malaria illness (Table 1). No significant differences were observed for admission characteristics of women enrolled and not enrolled in the fever (data not shown).

**Table 1:** Characteristics at baseline of febrile pregnant women with malaria and non-malaria morbidity.

Characteristic		Non-malaria morbidity	Malaria	Non malaria morbidity and Malaria	P^a^
		N = 171	N = 227	N = 11	
Refugee women, N (%)		114 (66.7)	55 (24.2)	2 (18.2)	**<0.001**
Age, mean±SD, years		27±7 [15–41]	26±7 [15–46]	27±7 [16]–[35]	0.568
Gravidity		3 [1]–[12]	3 [1]–[12]	4 [1]–[9]	0.83
Parity		1 [0–9]	1 [0–11]	3 [0–5]	0.85
Primigravida, N (%)		46 (26.9)	67 (29.5)	3 (27.3)	0.577
Smokers, N (%)		46 (27.4)	100 (44.4)	4 (36.4)	**0.001**
History of fever, days		2 [1]–[21]	3[1]–[10]	2 [1]–[6]	0.202
Fever >3 days, N (%)		36 (24.3)	50 (24.0)	1 (9.1)	1.000
Mean gestation, wks		23± 9 [5–41]	21± 10 [4–41]	20± 12 [6]–[38]	**0.030**
Trimester of pregnancy, N (%)	1st	30 (17.5)	65 (28.6)	4 (36.4)	**0.014** b
	2nd	82 (48.0)	101 (44.5)	4 (36.4)	
	3rd	59 (34.5)	61 (26.9)	3 (27.2)	
Temperature, °C		38.3 [37.6–40.5]	38.3 [37.6–40.6]	38.5 [37.9–39.5]	0.995

Data are mean ± SD [range] where SD is standard deviation or proportion N (%).

a Comparison between non-malaria and malaria group only.

b Chi-square test for linear trend.

### Detailed fever investigation cohort

The following results concentrate on the 203 women who had further investigation for fever (Figure 2). A total of 211 potential etiologies of fever were identified (Table 2) i.e. 8 women (3.9% (8/203)) had two, laboratory confirmed diagnoses and these were mainly with malaria (7/8) (Table 3). Of women with malaria, rickettsial coinfection occurred in 9.8% (95%CI: 4.2–20.0) (5/51) of cases. Arthropod (malaria, rickettsial infections, and dengue) and zoonotic disease (leptospirosis) accounted for nearly half of all febrile illnesses in pregnant women, 47.3% (96/203). Most (82% (99/121) refugee camp women opted for HIV testing and 3% (3/99) were HIV positive (2 of these women had CD4 T lymphocyte counts less than 250/mm^3^).

**Table 2:** Number of diagnoses (N = 211) in pregnant women (N = 203) with fever.

DIAGNOSIS	N	%
Malaria	51	24.2
*P.falciparum* n = 28		
Mixed (*P.falciparum* & *P.vivax*) *n = 3*		
*P.vivax* n = 20		
Pyelonephritis	41^c^	19.4
urine culture positive n = 32^a^		
urine culture negative n = 9 b		
(Asymptomatic Urinary Tract Infection)	1	0.5
Acute undifferentiated febrile illness	40	19.0
Rickettsia^d^	26	12.3
Scrub typhus n = 11		
Murine typhus n = 14		
Murine+Scrub typhus n = 1		
Dengue	20	9.5
Acute respiratory infection e	17	8.1
Leptospirosis	5	2.4
Enteric fever^f^	2	0.9
Suspected chorioamnionitis	2	0.9
Endometritis^g^	2	0.9
Chicken pox	1	0.5
Cholecystitis	1	0.5
Gastroenteritis	1	0.5
Pyelolithiasis	1	0.5
**TOTAL**	**211**	**100**

a Organisms cultured from urine were *Escherichia coli* 87.9% (29/33), and one each of *Citrobacter* sp, *Enterococcus* sp., *Klebsiella* sp. and *Klebsiella oxytoca*.

b 7 of these were pre-treated with antibiotics before urine culture.

c 8 of these women also had *E. coli* bacteraemia.

d Confirmation of typhus was by serology in 9 women, serology and PCR in 17 women (murine 7, scrub 9, both 1) and in 3 cases *O. tsutsugamushi* was isolated from culture (PCR and serology were also positive.).

e 7 upper tract infection and 10 lower tract infection.

f 2 *Salmonella typhi* bacteraemia.

g 1 of which was *Klebsiella spp*. Bacteraemia.

**Table 3:** Laboratory confirmed diagnosis in women with coinfection.

Diagnosis 1	Diagnosis 2	N	Rickettsia confirmation method
*P.falciparum*	Murine Typhus	2	Dynamic serology
*P.falciparum*	Scrub Typhus	2	PCR
*P.vivax*	Murine Typhus	1	PCR
*P.falciparum*	Asymptomatic urinary tract infection	1	NA
Mixed (*P.falciparum* & *P.vivax*)	Dengue	1	NA
Pyelonephritis	Dengue	1	NA
TOTAL		8	

NA not applicable.

### Clinical predictors of diagnosis

The most common symptoms on admission in order of frequency were: headache 89.7% (182/203), anorexia 74.9% (152/203), muscle pain 64.0% (130/203), joint pain 58.6% (119/203) and dizziness 56.2% (114/203). Median duration of fever and proportion of women with more than 3 days fever were highest with scrub typhus at 5 [1]–[10] days, 63.6% (7/11 women); and lowest in women diagnosed with coinfection (2 [1]–[3] days; 0/8 women, P<0.05) and in dengue fever (2 [1]–[4] days and 5.6% (1/18 women) P<0.05).

Dysuria and costovertebral angle tenderness were significantly more common in women with pyelonephritis: reported in 65.0% (26/40) and 87.5% (39/40), (P<0.001, for both). Eschar 18% (2/11), and maculopapular rash 18% (2/11) were evident in three women (one had both) and were highly specific for scrub typhus. Petechial rash on admission was reported in only one case of acute undifferentiated febrile illness.

Clinical prediction of the diagnosis before any laboratory test results were known was poor except for pyelonephritis. The proportion of cases where the physician correctly predicted the diagnosis (excluding malaria) were 22.4% (4/18) of dengue, 19% (4/21) of rickettsial infections, 16.7% (1/5) of leptospirosis, and 50% (1/2) of enteric fever (but only because the husband was admitted with typhoid to another hospital).

### Complete blood count as a diagnostic indicator

**Figure 3:**
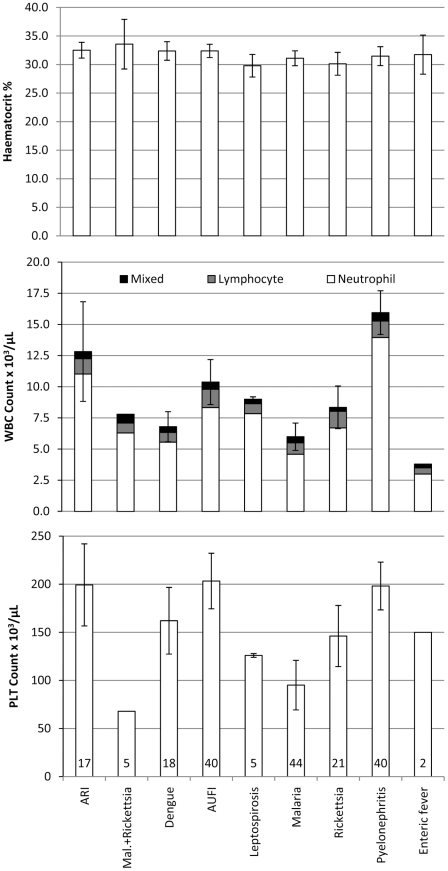
Complete blood count results (mean and 95% CI) by diagnostic group. WBC – white blood cells, PLT – platelet, Mal. – malaria. Number at base of platelet column is the sample size for each diagnostic group.

The complete blood count was available for 77.8% (158/203) of women on enrolment (Figure 3). There was no significant difference in haematocrit values (P = 0.53). Compared to other diagnoses the mean white cell and neutrophil counts were higher in women with pyelonephritis and acute respiratory illness (P<0.05) and the mean platelet count lower in women with malaria and malaria plus rickettsial infection (P<0.05). Women with enteric fever had the lowest mean white cell count but the sample size was only two. Complete blood count was not more discriminatory for rickettsia, dengue, or leptospirosis in this cohort.

### Birth outcome and neonatal death

Overall 8.4% (17/203) of women were lost to follow-up before the birth outcome was known. The proportions of lost to follow-up (8.4% (17/203) overall) and miscarriage (10.2% (19/186) overall) were similar across the diagnostic categories (Table 4), (P = 0.623, P = 0.15). Of the 167 births the stillbirth rate was 24.0 (95%CI 9.4–60.0) per 1,000 births. The stillbirths were not caused by intrapartum labour problems. Three of the four stillbirths occurred in the rickettsial infection group (two scrub (one PCR confirmed) and one murine (PCR confirmed)). The congenital abnormalities identified in 6 newborns were unlikely to be related to the febrile illness based on the estimated gestation at the time of the fever episode (13.5 to 41.0 weeks).

**Table 4:** Outcome of pregnancy in selected diagnostic groups.

Diagnostic group, N	Known outcome	Miscarriage	Stillborn	CA	Mean EGA wks	Premature<37 wk	Weighed within 72 hrs birth	Mean birthweight g	LBW< 2500 g
AUFI	35	3	1	3	38.6±2.0	4	25	3000±474	3
40	(87.5)	(8.6)	(3.1)	(9.4)	[33.0–42.6]	(12.5)	(89.3)	[1950–3900]	(12)
ARI	16	1	0	0	39.2±1.3	1	14	3161±515	1
17	(94.1)	(6.3)			[36.3–41.2]	(6.7)	(93.3)	[2130–4000]	(7.1)
Dengue	18	1	0	1	39.4±1.6	1	15	2961±247	0
18	(100)	(5.6)		(5.9)	[34.4–42.1]	(5.9)	(93.8)	[2500–3430]	
Leptospirosis	5	0	0	0	40.2±0.9	(0)	5	3148±580	0
5	(100)				[39.1–41.2]		(100.0)	[2500–4050]	
Malaria and rickettsia	5	2	0	0	38.9±0.3	(0)	3	2820±166	0
5	(100)	(40.0)			[38.6–39.2]		(100.0)	[2700–3010]	
Malaria	37	5	0	1	39.5±1.4	3	26	2932±436	5
44	(84.1)	(13.5)		(3.1)	[36.0–41.4]	(9.4)	(83.9)	[2000–3750]	(19.2)
Pyelonephritis	37	2	0	1	38.9±2.5	3	33	2988±577	4
40	(92.5)	(5.4)		(2.9)	[29.2–42.5]	(8.6)	(97.1)	[1280–4060]	(12.1)
Murine typhus	10	0	1	0	38.7±2.4	2	9	2692±530	3
11	(90.9)		(10)		[34.2–41.5]	(20.0)	(100.0)	[1770–3260]	(33.3)
Scrub typhus	9	1	2	0	37.8±1.3	2	4	2810±787	2
9	(100)	(11.1)	(25)		[36.0–40.3]	(25.0)	(66.7)	[2200–3950]	(50)

Proportion are presented as N (%); Normal data as mean ± SD [range] where SD is standard deviation.

CA congenital abnormality, EGA estimated gestational age at birth; LBW low birth weight.

AUFI - Acute undifferentiated febrile illness; ARI – Acute respiratory tract infection.

Gestation at birth (P = 0.18), the proportion of premature infants (P = 0.87), the mean birth weight (P = 0.59) and the proportion of low birth weight infants (P = 0.16) did not differ between groups. However the diagnosis of rickettsial infection (pooled) versus all other etiologies was significantly associated with low birth weight (P = 0.006). Factors associated with low birth weight were determined by univariate analysis (Table 5). The only significant and independent risk factor for low birth weight (n = 144 live-born singletons) was estimated gestational age at delivery: OR 2.6 (95%CI: 1.7–3.9), P<0.001). In order to understand other contributing factors the regression model was run without including gestation at birth: rickettsial infection; OR 4.1 (95%CI: 1.1–14.8), P = 0.031; smoking; OR 3.3 (95%CI: 1.1–10.0), P = 0.039; and fever >3 days; OR 1.3 (95%CI: 1.03–1.7), P = 0.027; remained significant and independent predictors of low birth weight (Table 5). Most live-born infants were followed to 1 month of age 95.1% (155/163) and there was one neonatal death (day 12 of life, neonatal sepsis).

**Table 5:** Factors associated with low birth weight on univariate and regression analysis.

Factor	Factor present	Proportion low birth weight	P	Regression including prematurity in the modelß (95%CI)	Regression excluding prematurity from the modelß (95%CI)
Smoking	yes	22.9 (8/35)	**0.040**	NS	**3.5 (1.1**–**11.0)**
	no	9.3 (9/97)			
Primigravida	yes	20.5 (8/39)	0.158	NS	NS
	no	10.5 (10/95)			
History fever >3 days^a^	yes	24.1 (7/29)	0.056	NS	**1.3 (1.0**–**1.7)**
	no	10.5 (11/105)			
Infection 2^nd^ and 3^rd^ trimester^a^	yes	16.2 (18/111)	0.042	NS	NS
	no	0 of 22			
Prematurity^a^	yes	90.0 (9/10)	**<0.001**	**2.5 (1.6**–**3.8)**	NA
	no	7.3 (9/124)			
Rickettsial infection^b^	yes	38.5 (5/13)	**0.006**	NS	**5.3 (1.4**–**20.1)**
	no	11.0 (13/118)			

NS non significant, NA not applicable.

a History of fever >3 days, infection 2nd and 3rd trimester and premature delivery were included in the model as continuous variables.

b Only including women with rickettsial monoinfection.

### Maternal death

In this fever cohort there was one maternal death. The woman who died was a 33 year old (gravidity three, parity two), chronic asthmatic and smoker of 23.3 weeks gestation who presented with a one week history of fever. She died within 15 hrs of admission from suspected septic shock. All laboratory tests were negative. No post-mortem was done.

## Discussion

Febrile episodes occurred in approximately 5% (409/8,117) of pregnant women during the 29 months (2004–2006) of this study and 55.5% (227/409) of febrile women had malaria. Despite the marked reduction in incidence on the western border of Thailand [19], malaria remains the leading cause of fever in pregnancy in this low transmission area [20]. The overall proportion of women affected by febrile malaria episodes is likely to be reduced by the system of frequent antenatal clinics with active detection and treatment, allowing early diagnosis before women become symptomatic [2].

The true incidence of infections cannot be estimated from this study as the women with fever who did not enroll in the study did not have the full complement of laboratory investigations for fever. As the demographics of women with fever and malaria who did and did not enroll in the fever study were not significantly different the proportions are unlikely to be extremely biased. Arthropod borne (malaria, rickettsial infections, and dengue) and zoonotic (leptospirosis) disease accounted for nearly half of all febrile illness in pregnant women. Approximately 10% of women with malaria in this cohort had rickettsial coinfection. This differs to previously published data in non-pregnant adults presenting with fever 150 km south of Mae Sot on the Thai-Burmese border (1999–2002) where malaria was commonly involved in coinfection (17% of patients) but usually with leptospirosis [21]. That particular study tested for scrub typhus using IFA [22] however the samples processed were confined to 46 of the 613 enrolled patients selected using clinical criteria and hence may have underestimated malaria and rickettsial coinfection rates (only 2 such cases were described). The risk of coinfection may be site-specific and studies that specifically include patients with malaria are required to verify this finding.

Microscopy of malaria blood smear and urine sediment allowed diagnosis of the cause of fever in 44.8% (91/203) of women. For these infections there was little to be gained from performing further investigations such as complete blood count [23]. Clinical diagnosis of leptospirosis, dengue and rickettsial infections was completely unreliable.

Diagnostic and treatment algorithms would be useful in this setting. This study suggests that once malaria, pyelonephritis and acute respiratory infection have been excluded by microscopy and/or clinical findings, at least one third of the remaining infections will be due to rickettsia or leptospirosis- both of which require antibiotic treatment. A policy of parenteral ceftriaxone, metronidazole and azithromycin for severely ill febrile pregnant women and azithromycin alone for ambulant febrile women could be considered [24]. While doxycycline is the drug of choice in non-pregnant patients [25], [26], azithromycin is safer and as effective an option (albeit more expensive) in pregnant women for treatment of scrub typhus and leptospirosis [27]. In the severely ill woman ceftriaxone is a broad spectrum, well-tolerated antibiotic with good pharmacokinetic properties for pregnancy [28], [29], [30], [31]. A careful search for eschar [32] and rash will assist diagnosis.

Can these arthropod-borne infections be prevented? Although 90% of pregnant women use insecticide treated bednets they did not prove to be significantly protective against malaria infection in a randomized controlled trial conducted in this area in pregnant women [33]. Vector avoidance in this area is particularly difficult and not very amenable to change: women go to the forest for vegetables, work in the agriculture sector, and the houses are impossible to mosquito proof as they are constructed from bamboo. Skin repellents and mosquito deterrents are beyond the finances of the majority of the population.

There are limitations to the information presented in this manuscript. There was no radiology for respiratory infection. HIV testing was available for less than half of all women. The sensitivities and specificities of PCR, organism isolation and serology, change with the number of days of fever at which the patient presents and this may have affected the proportions observed. The strict serological criteria excluding women who did not show a 4 fold rise in antibody titre may have resulted in under-estimates of the true proportion of infected women for leptospirosis, dengue and rickettsial infections. Birth outcomes were known for a high proportion of women who entered the cohort but the small sample size by diagnostic group calls for cautious interpretation of the results. The poor birth outcomes in women diagnosed with rickettsial illness is a cause for concern but the numbers available for analysis were small and results of borderline significance. Stillbirths and low birth weight have been reported previously with rickettsial illness and less often for women with murine typhus [34], [35], [36], [37], [38]. No autopsy was available for the maternal death or stillbirth cases.

Arthropod borne fever in pregnancy contributes to maternal death and poor birth outcomes in resource poor tropical countries. As a result of improved malaria control with deployment of artemisinin combination therapy, long lasting insecticide treated nets and indoor residual spraying, the relative importance of non-malaria fever in pregnant women is likely to rise. The burden of HIV mortality and morbidity in pregnancy has been highlighted previously for African women [39], [40]. It also appears to be a risk factor for febrile morbidity in Asian pregnant women. Microscopy remains the most useful tool in the field for fever diagnosis in pregnant women for malaria and urinary tract infection. Leptospirosis, dengue and rickettsial infections require improved field based diagnostics to ensure that women receive appropriate antibiotic therapy.

## Supporting Information

Checklist S1 STROBE Checklist
(0.08 MB DOC)
