# Supplementary material for: Arthropod Borne Disease: The Leading Cause of Fever in Pregnancy on the Thai-Burmese Border
Source: PLoS Negl Trop Dis. 2010 Nov 16;4(11):e888. doi: 10.1371/journal.pntd.0000888 (PMC2982829; doi:10.1371/journal.pntd.0000888)
Supplement: Checklist S1 — STROBE Checklist (0.08 MB DOC) [file pntd.0000888.s001.doc]

STROBE Statement—Checklist of items that should be included in reports of ***cohort studies***

|  | Item No | Recommendation |
| --- | --- | --- |
| **Title and abstract** | 1 | (a)Indicate the study’s design with a commonly used term in the title or the abstract  *Line 37* |
|  |  | (*b*) Provide in the abstract an informative and balanced summary of what was done and what was found  *Line 29-45* |
| Introduction | | |
| Background/rationale | 2 | Explain the scientific background and rationale for the investigation being reported  Line 70-87 |
| Objectives | 3 | State specific objectives, including any prespecified hypotheses  *Line 85-87* |
| Methods | | |
| Study design | 4 | Present key elements of study design early in the paper  *Line 111-137* |
| Setting | 5 | Describe the setting, locations *(Line 97-104)*, and relevant dates , including periods of recruitment (*Line 113-114*), exposure (N.a) , follow-up(*Line 231-241; 244-45*), and data collection |
| Participants | 6 | (*a*) Give the eligibility criteria (*Line 116-124)*, and the sources and methods of selection of participants (*Line 113-114*). Describe methods of follow-up (*Line 217-245*) |
|  |  | (*b*) For matched studies, give matching criteria and number of exposed and unexposed N.A. |
| Variables | 7 | Clearly define all outcomes, exposures, predictors, potential confounders, and effect modifiers. Give diagnostic criteria, if applicable *(Line 140-215)* |
| Data sources/ measurement | 8* | For each variable of interest, give sources of data and details of methods of assessment (measurement) (*Line 142-245*). Describe comparability of assessment methods if there is more than one group. N.A. |
| Bias | 9 | Describe any efforts to address potential sources of bias N.A. |
| Study size | 10 | Explain how the study size was arrived at (*Line 112-114*) |
| Quantitative variables | 11 | Explain how quantitative variables were handled in the analyses. *(line 250-252)* If applicable, describe which groupings were chosen and why N.A. |
| Statistical methods | 12 | (*a*) Describe all statistical methods (*line 248-246*) , including those used to control for confounding |
|  |  | (*b*) Describe any methods used to examine subgroups and interactions (*line 252-256*) |
|  |  | (*c*) Explain how missing data were addressed NA |
|  |  | (*d*) If applicable, explain how loss to follow-up was addressed N.A. |
|  |  | (*e*) Describe any sensitivity analyses N.A. |
| Results | | |
| Participants | 13* | (a) Report numbers of individuals at each stage of study—eg numbers potentially eligible, examined for eligibility, confirmed eligible, included in the study, completing follow-up, and analysed (*Line 259-289*) |
|  |  | (b) Give reasons for non-participation at each stage N.A. |
|  |  | (c) Consider use of a flow diagram (*Figure 2*) |
| Descriptive data | 14* | (a) Give characteristics of study participants (eg demographic, clinical, social) and information on exposures and potential confounders (Line 259-284) |
|  |  | (b) Indicate number of participants with missing data for each variable of interest |
|  |  | (c) Summarise follow-up time (eg, average and total amount) N.A. |
| Outcome data | 15* | Report numbers of outcome events or summary measures over time (*line 287-295*) |
| Main results | 16 | (*a*) Give unadjusted estimates and, if applicable, confounder-adjusted estimates and their precision (eg, 95% confidence interval). Make clear which confounders were adjusted for and why they were included N.A. |
|  |  | (*b*) Report category boundaries when continuous variables were categorized *(table 4)* |
|  |  | (*c*) If relevant, consider translating estimates of relative risk into absolute risk for a meaningful time period N.A. |
| Other analyses | 17 | Report other analyses done—eg analyses of subgroups and interactions, and sensitivity analyses (*Line 298-353*) |
| Discussion | | |
| Key results | 18 | Summarise key results with reference to study objectives (*Line 356-377*) |
| Limitations | 19 | Discuss limitations of the study, taking into account sources of potential bias or imprecision. (*Line 404-416)* Discuss both direction and magnitude of any potential bias N.A. |
| Interpretation | 20 | Give a cautious overall interpretation of results considering objectives, limitations, multiplicity of analyses, results from similar studies, and other relevant evidence (*Line 369-377*) |
| Generalisability | 21 | Discuss the generalisability (external validity) of the study results (*Line 418-425*) |
| Other information | | |
| Funding | 22 | Give the source of funding and the role of the funders for the present study and, if applicable, for the original study on which the present article is based “manuscript data” |

*Give information separately for exposed and unexposed groups.

**Note:** An Explanation and Elaboration article discusses each checklist item and gives methodological background and published examples of transparent reporting. The STROBE checklist is best used in conjunction with this article (freely available on the Web sites of PLoS Medicine at http://www.plosmedicine.org/, Annals of Internal Medicine at http://www.annals.org/, and Epidemiology at http://www.epidem.com/). Information on the STROBE Initiative is available at http://www.strobe-statement.org.
